# Communal Innovations: Inspiring Neighborhoods of Hope and Advocacy

**DOI:** 10.1080/10810730.2020.1785059

**Published:** 2020-07-02

**Authors:** Rachel A. Smith, Youllee Kim, Stephen A. Matthews, Eleanore D. Sternberg, Dimi Théodore Doudou, Matthew B. Thomas

**Affiliations:** 1 Department of Communication Arts and Sciences, The Pennsylvania State University, University Park, PA, USA; 2 Department of Sociology and Criminology and the Department of Anthropology, The Pennsylvania State University, University Park, PA, USA; 3 Department of Entomology, The Pennsylvania State University, University Park, PA, USA; 4 Centre de recherche pour le Développement (CRD)/Laboratoire de Santé, Nutrition et Hygiène, Université Alassane Ouattara, Bouaké, Côte d’Ivoire

## Abstract

Innovations promise a better future, which may generate feelings of hope and inspire advocacy. Some innovations are more communal in nature: attempting to address a social problem, through community engagement and wide-spread adoption. For such innovations, the social processes that involve collective aspects of community life may play important roles in fostering hope and interpersonal advocacy. This study uses communication infrastructure theory and discrete emotions theory to investigate hope and advocacy within a field trial for a salient, visible, community-bound innovation to reduce transmission of malaria. Heads of households in one community (*N* = 119) in West Africa were interviewed. Results showed that innovation hope was predicted by appraisals of innovation attributes. Better appraisals of the innovation’s attributes, greater perceived collective efficacy, and recent malaria illness predicted more innovation advocacy. The spatial analysis showed that innovation advocacy was geographically clustered within the community, but hope was not. The implications for theory and practice are discussed.

Hope is the thing with feathers/That perches in the soul,/And sings the tune without the words,/And never stops at all.

–—Emily Dickinson, 1891

In her poem, Emily Dickinson provides the image of hope as a bird that survives any storm and continues to sing. This is not always true: people can lose hope, and communities can become hopeless in the face of chronic, devastating threats to wellbeing. Recently we have learned much about how communication can activate the cognitions that inspire hope, and how hope motivates positive behaviors (Chadwick, 2015; Nabi & Myrick, 2018). Extant hope research has focused primarily on individuals: personal appraisals of problems and personal action to address them. Yet, sometimes social units—groups, neighborhoods, communities—experience problems that affect the common good. Finding ways to inspire hope about collective problems may be an important way to promote collective action and community engagement.

Sometimes, problems need to be addressed with innovations: new ideas, technologies, or practices (Rogers, 2003). For collective issues, diffusion may be especially important, because some innovations may need to be adopted throughout a community to be effective. Rogers (2003) emphasized how diffusion is based in communication: as people discuss novel ideas, technologies, or behaviors, their discussions can foster systemic social change. And yet, these communication activities—such as interpersonal advocacy—have received little attention. Furthermore, emotional mechanisms, such as hope, have received no theoretical attention.

In this study, we explored the idea that for communal innovations (i.e., innovations framed as collective efforts to address social problems) the social processes of community life may foster hope and interpersonal advocacy. Further, we explored the degree to which hope and advocacy are geographically clustered within a community. To frame this study, we drew upon communication infrastructure theory (CIT; Kim & Ball‐Rokeach, 2006a) and theories of discrete emotions (e.g., Lazarus, 1991). The context of this study is a field trial for a salient, visible, community-bound innovation to reduce transmission of malaria in West Africa. To begin, we consider innovation attributes and hope.

## Hopeful Innovations

Innovations should be a prime persuasive target for generating hope, because innovations promise a better future (Dearing, 2009). Indeed, most persuasive communication to promote innovations addresses how they will fulfill that promise (e.g., being more effective, cheaper, easier, pleasurable, prestigious, and less risky than previous options; Rogers, 2003) because such innovations are more likely to be adopted.

One way to conceptualize hope is as a discrete emotion: a categorical emotional state with unique causes, subjective experiences, and action tendencies (Nabi, 2010). Theories of discrete emotions (e.g., Lazarus, 1991) claim that as people monitor the world around them, specific appraisals of the environment cause them to experience particular (discrete) emotions (Lazarus, 1991; Roseman, 1991). The appraisals leading to hope are (a) assessing a problem as important to resolve (Chadwick, 2015), and (b) uncertainty as to whether positive resolution will occur (Chadwick, 2015; Roseman, 1991). Situational uncertainty is key: hope arises “when the odds do not greatly favor it” (Lazarus, 1991, p. 282). Hope also involves imagination: “To hope is thus to conjure in one’s imagination a picture of the world in which the objective of one’s hope has been realized” (Webb, 2007, p. 73). The subjective feeling of hope is pleasant and mildly arousing (Lazarus, 1999); it is described as a feeling of anticipation, readiness, and eagerness to realize the desired outcome (Roseman, 2001).

As people feel hope, they engage in future, goal-directed action (Lazarus, 1991, 1999; Snyder, 2000), especially when facing hardship (Averill, Catlin, & Chon, 1990; Bruininks & Malle, 2005; Lazarus, 1991; Nabi & Myrick, 2018; Stotland, 1969). Hope has become a focus for health communication researchers trying to encourage individuals to engage in prolonged action. For example, patients recovering from surgery who can imagine their full recovery may feel hope that, in turn, motivates them to engage in difficult and lengthy physical therapy.

We argue that the innovation attributes associated with adoption in diffusion research (Rogers, 2003) may do so because they generate hope. Rogers (2003) notes five innovation attributes associated with adoption: relative advantage (i.e., innovation is better than what currently exists), compatibility (i.e., it is consistent with existing values, past experiences, and adopters’ needs), complexity (i.e., it is easy to understand and use), trialability (i.e., it can be experimented with before adoption occurs), and observability (i.e., results are visible). These innovation attributes promise a better future, matching core appraisals associated with evoking hope. To contextualize these abstract concepts, we describe a large-scale field trial of a novel vector control—the Screening and Eave Tubes (SET) innovation—in Cote d’Ivoire, West Africa.

## SET Innovation: Case Study for Investigation

Despite decades of intervention, few countries have eliminated malaria (World Health Organization, 2017). In 2016, 90% of the 216 million people with malaria lived in Africa (World Health Organization, 2017). Reducing malaria is an important topic for Cote d’Ivoire (Sternberg et al., 2018). Using insights into mosquito and human behavior, researchers created a suite of home modifications, the SET innovation, which includes closing points of entry for mosquitoes into the home (e.g., closing eaves); adding barriers to windows and doors (e.g., installing window screening), installing In2Care EaveTubes into the eaves; and placing an insert with insecticide into the tubes (for details, see Knols et al., 2016).

Regarding its innovation attributes, the SET innovation has relative advantages over existing methods to limit indoor mosquitoes, strong compatibility with needs for indoor comfort, and low complexity (once installed, there is little maintenance required). The SET innovation does not have trialability: The house modifications and installation of the EaveTubes are difficult to undo. The innovation may also have limited observability: People are unlikely to see mosquitos dying or find dead mosquitoes, because the mosquitoes typically fly off before they die. However, the SET innovation itself is visible: The sealed walls and tubes are noticeable and readily observable. The innovation may be perceived as making the house more attractive.

Importantly, innovations are inherently uncertain; the primary role of diffusion—as a communication activity—is to reduce that uncertainty (Rogers, 2003). For the SET innovation, there is uncertainty about whether it will work in a non-experimental setting (i.e., a larger community), and how it will affect community participants. The context of a field trial increases the salience of uncertainty about the innovation, because that is what the trial attempts to assess (Smith, Morrow, & Ross, 2015). This uncertainty sets the stage for the appraisal process that leads to hope. Assuming malaria is important to address, as people appraise the SET innovation as having stronger innovation attributes, they should feel more hopeful about it.

*H1*: Stronger appraisals of the innovation’s attributes predict greater hope.

### Role of SET Failure: Recent Malaria Sickness

This study was conducted one year after the innovation was installed in participants’ homes. Based on diffusion research (Rogers, 2003), we would expect that the experience of recent malaria sickness could be perceived as the innovation’s failure to fulfill its promise to provide a future without malaria.

*H2*: People who have been ill with malaria in the past two weeks, versus those who have not, will report less hope for reducing malaria.

## Communal Aspects of the SET Innovation

In addition to providing hope for a future without malaria, the SET innovation is a communal innovation, because (a) it addresses an environmental issue that affects the community’s health (mosquito-borne malaria), (b) the SET innovation needs wide-spread adoption to succeed, and (c) the field trial was designed as a community event.

The SET innovation was predicted to provide community protection at a certain threshold: at least 65% of homes (Waite, Lynch, & Thomas, 2016). Screening alone was expected to benefit individual householders, but the real benefits of the SET innovation for the community could only be achieved with wide-spread adoption. The goal of the SET innovation was not just to provide protection to individual homeowners, but to protect those who did not adopt the technology as well by lowering the problematic population of mosquitos from the entire geographic area (Waite et al., 2016). This is not unique to the SET innovation: Many innovations to reduce infectious mosquito populations need to be carried out by enough people within a geographic area to affect the mosquito population (Hawley et al., 2003; Killeen & Moore, 2012; Waite et al., 2016).

These projects are often promoted through community-wide activities, such as town-hall meetings or media coverage, to gain collective approval and action (Adhikari et al., 2016; Sahan et al., 2017; Whittaker & Smith, 2015). The field trial for the SET innovation engaged the community in a few ways (see Sternberg et al., 2018 for details). Researchers obtained community leaders’ permission to talk to the community, then held a town-hall meeting in each village in the local “communication hotspot” (Wilkin, Stringer, O’Quin, Montgomery, & Hunt, 2011, p. 203) to explain the SET innovation and the trial protocol. The town-hall meeting included a video (in Baoulé) that explained the EaveTubes and house modifications and a description of the project activities (e.g., random assignment of communities and epidemiological monitoring).

A few days after the town hall, the community leader was contacted to ascertain whether the community agreed to participate; if they agreed, community leaders were invited to meet at the research center, where they learned about randomization. Last, communities in the ‘treatment’ arm lived through a big, public construction project in suitable homes. This study investigated one community in the intervention arm of the field trial. To understand innovation advocacy as facet of community problem-solving and capacity building, we turned to CIT (Kim & Ball-Rokeach, 2006b; Kim & Ball‐Rokeach, 2006a), which is described next.

## Communication Infrastructure Theory

Social systems shape diffusion (Rogers, 2003). The presence of opinion leaders and change agents and social norms can propel or inhibit conversations about innovations. Although diffusion research considers social systems important, it does not theorize why some communities have greater capacity for addressing adversity. CIT (Kim & Ball-Rokeach, 2006b; Kim & Ball‐Rokeach, 2006a) argues that the communication fabric of a community plays a critical role in shaping a community and its capacity for civic engagement. According to CIT, communities have storytelling systems, which are comprised of interactions within and across macro-, meso-, and micro-level actors (e.g., mainstream media, community organizations, and interpersonal interactions among neighbors, respectively). The storytelling network enables communities to build a shared discourse: shared desires, lived experiences, obstacles, and solutions. The “stories are the building blocks of the ability to ‘imagine’ an area as a community” (Kim & Ball‐Rokeach, 2006a, p. 178).

CIT describes collective efficacy as a means by which people internalize the local stories gathered through a neighborhood storytelling network into their everyday lives (Kim & Ball‐Rokeach, 2006a). According to CIT, to produce action, people must internalize the neighborhood storytelling network in positive ways, such as collective efficacy, which refers to “individuals’ perceptions of whether neighbors will join together to solve neighborhood problems” (Kim & Ball‐Rokeach, 2006a, p. 188). A strong storytelling network has been associated with high levels of collective efficacy (Kim & Kang, 2010; Matsaganis, 2008; Matsaganis & Wilkin, 2015). Wilkin’s (2013) review of CIT research noted the importance of micro-level storytelling—the interpersonal conversations between neighbors— in predicting civic engagement and health outcomes, through their impact on internalized beliefs, such as collective efficacy. With our emphasis on micro-level connections in this study, we focused our prediction as follows:

*H3*: More micro-level neighborhood storytelling predicts greater collective efficacy.

Studies framed by CIT have explored the role of storytelling networks on various outcomes, including disease knowledge (e.g., Kim, Moran, Wilkin, & Ball-Rokeach, 2011), disease outcomes (e.g., hypertension; Walter, Robbins, Murphy, & Ball-Rokeach, 2017), and preventative health behaviors (e.g., Wilkin, Katz, Ball-Rokeach, & Hether, 2015). Although existing studies have not considered emotion, the original theory does not exclude emotion. In fact, there is evidence that collective efficacy predicts emotions such as hope.

Recent research into the emotions tied to efficacy appeals suggests that self-efficacy (i.e., I can do it) and response-efficacy (i.e., the recommended behavior can address a problem) help people imagine a future with relief from adversity (Nabi & Myrick, 2018), while inability to imagine positive future results in sadness (Nabi & Myrick, 2018). Empirical research supports the positive relationship between efficacy (self and response) and hope (Nabi & Myrick, 2018). For innovations framed as collective efforts, collective efficacy is relevant (Bandura, 2000; Wlodarczyk, Basabe, Páez, & Zumeta, 2017). Thus, we explore the influence of the neighborhood storytelling network on people’s daily lives through collective efficacy by considering emotional outcomes, specifically hope about the SET innovation.

*H4*: Greater collective efficacy predicts greater hope for reducing malaria.

## Local Innovation Advocacy

According to CIT (Kim & Ball‐Rokeach, 2006a), the communication fabric of a community and how it is internalized by people living within the community shapes civic engagement. Also, CIT maintains that these generalized capacities and social cognitions need to foster dialogue about specific concerns or opportunities to generate the specific actions to address them. “When residents talk about their community in neighborhood council meetings, at a neighborhood block party, at the dinner table, or over the fence with neighbors, they become local storytelling agents—participants in an active imaging of their community” (Kim & Ball‐Rokeach, 2006a, p. 179). In this study, people have many opportunities to discuss the SET innovation with each other, encouraging or discouraging its use in the community. We refer to this form of civic engagement as innovation advocacy.

We considered several factors that might affect innovation advocacy. In CIT (Kim & Ball-Rokeach, 2006b), more serious problems can stimulate greater community engagement. Thus, we expected residents recently sick with malaria to be more likely to advocate for the innovation, because malaria presents as a greater problem to address. Further, based on the logic of CIT, as residents appraise the innovation as having better attributes and perceive greater collective efficacy in their community, we expected that they would advocate more for the SET innovation. Last, advocacy provides opportunity to rehearse the imagining of the better future; thus, we expected advocacy to generate hope.

*H5*: People who have experienced malaria in the past two weeks, versus those who have not, will report more advocacy of the SET innovation to neighbors and community leaders.

*H6*: Stronger appraisals of the innovation’s attributes and greater collective efficacy predict more advocacy for the SET innovation to neighbors and community leaders.

*H7*: People who advocated more for the SET innovation will report greater hope.

## Neighborhood Effects

**Figure 1.**
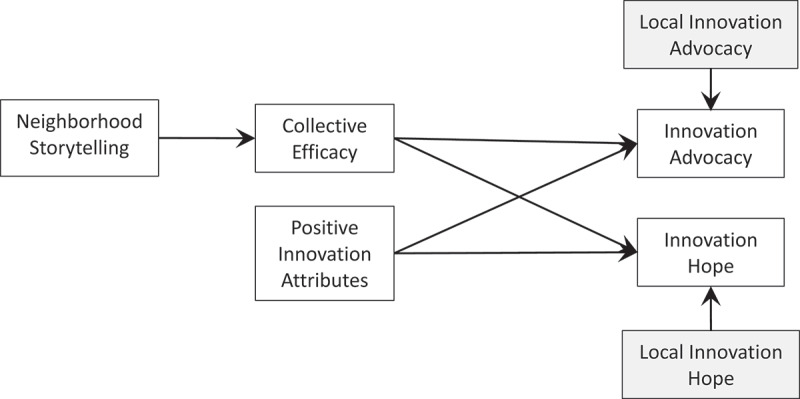
Theoretical model of situation appraisal (innovation attributes, issue importance, storytelling network, and collective efficacy) and advocacy effects on innovation-related hope.

Figure 1 shows the theoretical model of innovation hope and advocacy framed by CIT (Kim & Ball-Rokeach, 2006b; Kim & Ball‐Rokeach, 2006a) and theories of discrete emotions (e.g., Lazarus, 1991). These predictions suggest that living in the same neighborhood allows people to experience the same circumstances, and thus, report similar appraisals of events and community life and, ultimately, similar outcomes. Yet, these intrapersonal predictions do not fully embrace the spirit of CIT (Kim & Ball-Rokeach, 2006b; Kim & Ball‐Rokeach, 2006a), which presumes that neighbors influence each other through conversation, and this social influence shapes people’s cognitive and affective experiences. Previous studies of CIT have explicitly included spatial dependence—the tendency to be similar to those closer in geographic proximity—to capture these neighbor effects (e.g., Kim & Ball-Rokeach, 2006b). For communal interventions, even without interpersonal conversation, a shared experience creates a common fate in people’s experiences; using interdependent methods allows us to assess the type and degree of dependencies among observations within the system (Gonzalez & Griffin, 2000; Kenny, Mannetti, Pierro, Livi, & Kashy, 2002). Spatial analysis is particularly appropriate for processes that emphasize co-location, such as social norms, communication, and mimicry (Anselin, 1999).

Emotions and advocacy are processes that may create dependencies, but their patterns may differ. Emotions can be shared (Sullins, 1991) through interpersonal influence (e.g., influencing a neighbor’s appraisal; Parkinson, 2011; Parkinson & Simons, 2009) and through contagion (e.g., feeling hopeful because a neighbor looks hopeful, Hatfield, Cacioppo, & Rapson, 1993). Through both routes, emotions create clusters or neighborhoods of people sharing similar levels of hope. In contrast, communication may create asymmetry: as one person talks, others may listen (e.g., Bavelas, Coates, & Johnson, 2000, 2002). In line with CIT research, we explored potential spatial dependence in innovation hope and innovation advocacy.

*RQ1*: Does innovation hope have spatial dependence?

*RQ2*: Does innovation advocacy have spatial dependence?

## Methods

### Participants

Participants were the heads of household in one community assigned to the intervention arm of the field trial. The surveyors approached 137 homes; 87% agreed to participate (*N* = 119). The respondents (61% female, 36% male, 3% not reported) on average were 54 years old (*SD *= 15.42, *Mdn *= 54, *Mode *= 43). Fifty-three percent were married, 33% were widowed, and 15% were single. Respondents reported having no formal schooling (73%), having attended primary school (14%), secondary school (8%), or post-secondary school (4%); 90% reported working as plant farmers.

### Procedures

University institutional review boards and the Ministry of Health in Côte d’Ivoire approved this study. Data were gathered one year after the start of the SET field trial. Personal, face-to-face interviews were conducted using a structured questionnaire that contained open-ended and closed-ended questions. The questionnaire was developed in English, translated into French, and then back-translated into English by two independent bilingual translators (one translator was from the study area) to assess translation equivalence. The back-translation verified the accuracy of 95% of the content; 5% had issues in translation equivalence (e.g., definitions, grammar, idioms). The research team and translators met to resolve problematic content. The same procedure was conducted to translate the French version into Baoulé, a local language in the study villages. Baoulé is an oral language without written characters, but phonetic symbols can be used to write and read it. The questionnaire was filled out in French, but the interviewers also had a mirrored version open on the screen in Baoulé to minimize errors during the oral translation process. The three interviewers attended three, 8-hour training sessions to learn the standard translation, survey process, and technology usage.

Interviewers approached each household and asked to talk to the head of the household, described the study, and read the informed consent information, explaining that participation was voluntary and answers were confidential. Interviewers recorded participants’ responses on a tablet computer, using the Qualtrics off-line application. After entering the participants’ answers, interviewers recorded the alphanumeric ID code and geographic coordinates of the house.

### Measurement

A confirmatory factor analysis of the measurement model’s scales‒neighborhood storytelling network, collective efficacy, innovation attributes, local innovation advocacy, and innovation hope‒was estimated with maximum likelihood in AMOS (Version 25). The measurement model showed reasonable fit: χ^2^(339, *N* = 119) = 597.43, *p* <.05, SRMR = .08, RMSEA = .08, 90% CI [.07,.09]. Scale items were averaged; higher scores indicated more of the variable.

#### Neighborhood Storytelling Network

Five items (adapted from Kim & Ball-Rokeach, 2006b) were used to assess the local storytelling network (e.g., how often do neighbors speak about problems affecting this community?). Responses, marked on 5-point scales (1 = *never*, 5 = *all the time*), were averaged into one score (Cronbach’s α = .88).

#### Collective Efficacy

Six items (adapted from Kim & Ball-Rokeach, 2006b; Sampson, Raudenbush, & Earls, 1997) were used to assess collective efficacy (e.g., people around here are willing to help their neighbors). Responses, marked on 5-point scales (1 = *never*, 5 = *all the time*), were averaged into one score (Cronbach’s α = .84).

#### Innovation Attributes

Nine items (based on Rogers, 2003) were used to assess attitudes about the innovation’s attributes regarding relative advantage, compatibility, complexity, and observability (e.g., how much do you agree that the SET innovation is better than other malaria prevention technologies, is safe for children, fits with my lifestyle, will make my house prettier). Responses, marked on 5-point scales (1 = *not at all*, 5 = *very strongly*), were averaged into one score (Cronbach’s α = .83).

#### Local Innovation Advocacy

Four items (adapted from Boster, Carpenter, Andrews, & Mongeau, 2012) were used to assess the intensity of personal promotion about the SET innovation (e.g., I have encouraged people to install the SET innovation in their home). Responses, marked on 5-point scales (1 = *never*, 5 = *all the time*), were averaged into one score (Cronbach’s α = .97).

#### Innovation Hope

Four items (adapted from Chadwick, 2015; Nabi & Myrick, 2018) were used to assess the intensity of hopeful feelings about the SET innovation (e.g., I am hopeful that the SET innovation will reduce malaria). Responses, marked on 5-point scales (1 = *not at all*, 5 = *very strongly*), were averaged into one score (Cronbach’s α = .83).

#### Longitude and Latitude Information

Point-based geospatial data (longitude and latitude information about each household) were collected to examine spatial interdependence in observed variables.

## Results

### Descriptive Statistics

Means, standard deviations, and overall spatial clustering (global Moran’s *I*) appear in Table 1. As anticipated, respondents reported that preventing malaria was very important (*M* = 4.22, *SD *= 0.41); no one reported that preventing malaria was unimportant. On average, respondents reported an active neighborhood storytelling network, in which neighbors talk to each other and discuss problems affecting the community and ways to improve community life (*M* = 3.42, *SD *= 0.69). On average, respondents reported collective efficacy in their community to take care of one another, cooperate, and join together to solve community issues (*M* = 3.54, *SD *= 0.62).

**Table 1.** Descriptive statistics and global Moran’s I (N = 119)

	*M*	*SD*	Global Moran’s *I*
Neighborhood storytelling	3.42	0.69	−.03
Collective efficacy	3.54	0.62	−.04
Innovation attributes	4.01	0.59	.03
Recent malaria	−.65	0.77	−.01
Innovation advocacy	2.64	1.43	−.14*
Innovation hope	3.75	0.71	.01

*Notes*. The overall spatial clustering (global Moran’s *I*) was based on a distance-based spatial weights matrix (using a 60 m threshold). Recent malaria was effect-coded (1 = *sick with malaria in the past 2 weeks*; −1 = *not sick*); 17.6% reported being sick with malaria in the past 2 weeks.

*p <.05.

On average, regarding the SET innovation’s attributes, respondents perceived relative advantage over other methods, good compatibility with their lifestyle, low complexity, and house beautification (*M* = 4.01, *SD *= 0.59). Seventeen percent of the respondents reported experiencing malaria in the past two weeks. On average, respondents reported that they sometimes advocated for the innovation, such as recommending it to others and encouraging others to have it installed (*M* = 2.64, *SD* = 1.43). Respondents, on average, felt hopeful about the SET innovation’s ability to reduce malaria (*M* = 3.75, *SD *= 0.71).

In the community selected for this study (AUTHOR BLIND), 95% of homes received the SET innovation; similarly, in our sample 97% of respondents had SET installed in their homes (at no cost to the homeowner). Everyone in the sample reported seeing the SET innovation.

#### Global Spatial Autocorrelation

**Figure 2.**
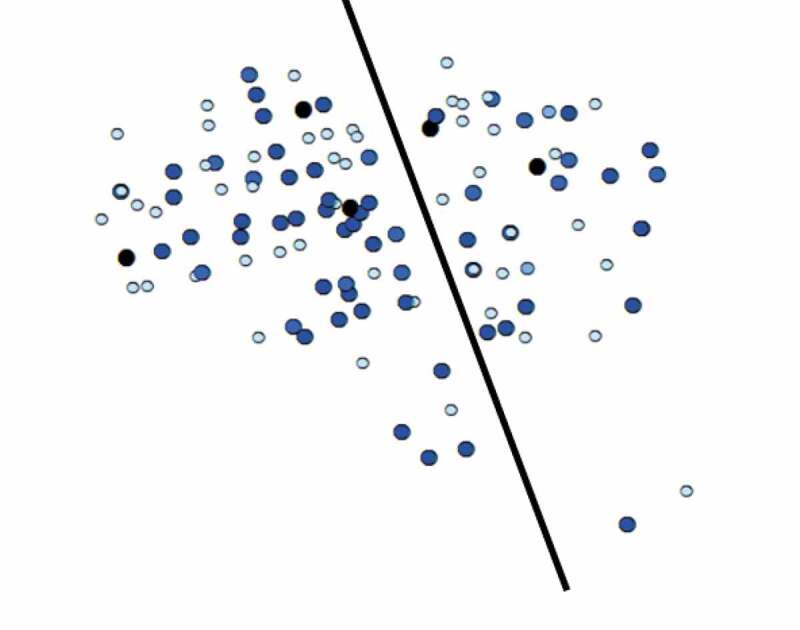
Visual depiction of the community. Participating households are marked with circles; the colors indicate each household’s level of innovation advocacy from light blue (never advocating) to dark blue (advocating all the time). The dark black line indicates a main road that cuts through the community.

Figure 2 is a visual representation of the community participants. The dark line shows the main thoroughfare cutting through the community. Geographic clustering is a key feature of this investigation. Global Moran’s *I* is an inferential statistic about the distribution of observed values in space; the null hypothesis of the Global Moran’s I is that the observed values are distributed at random in the study area (Anselin, 2003; Fischer & Getis, 2010). Global Moran’s *I* varies from 1 (perfectly clustered; e.g., very hopeful people next to very hopeful neighbors) to −1 (perfectly dispersed; e.g., very hopeful people next to very hopeless neighbors); 0 is interpreted as no systematic spatial pattern.

The global Moran’s *I* for each variable was calculated using GeoDa version 1.12, using a spatial weight matrix based on distance (see Table 1). The threshold distance was set as 0.06 km (60 meters), as this was the distance that ensures every household has at least one neighbor (*M* = 8.84, *SD *= 4.18, *Mode *= 8, *Median *= 9, *Minimum *= 1, *Maximum *= 22 neighbors). The community had a small footprint: 476 meters by 566 meters. The results showed low levels of overall clustering for the key variables. Only innovation advocacy was statistically significant: Global Moran’s *I* = −.14, *p* < .05. These results answered RQ1 and RQ2: innovation hope was not spatially clustered, but innovation advocacy showed dispersion (i.e., high advocators near low advocators).

### Hypothesis Testing

Due to interdependence in the data, all hypotheses were tested in GeoDa, which allowed us to estimate the models with ordinary least squares (OLS) and with a spatial lag model, which uses a maximum likelihood model with a spatially lagged dependent variable (Anselin, Syabri, & Kho, 2006).^1^^1^The model is *y* = *ρWy* + *Xβ* + ε, where *y* is a vector of observations on the dependent variable (i.e., innovation hope), *Wy* is a spatially lagged dependent variable for the weights matrix *W, X* is a matrix of observations on the explanatory variables, ε is a vector of i.i.d. error terms, and *ρ* and *β* are parameters (Anselin et al., 2006). The OLS model produces typical fit statistics for the regression model, such as an *F*; the spatial lag model does not. The fit indices that are produced for both models and used for comparison are the Akaike information criterion (AIC) and log-likelihood (LL).

#### Spatial Dependence

As a brief review, spatial dependence occurs when a person’s scores are interdependent with people in neighboring locations. Interdependence, particularly of dependent variables, violates the assumptions of OLS models. Dependent variables may be interdependent because a person’s dependent score is predicted by both their and their neighbor’s independent variables (i.e., spatial lag). This represents the sort of social process considered in this study, in which people shape the outcomes of others near them (neighbors). In GeoDa, OLS regression produces diagnostic tests to test for and identify the form of spatial dependence.

#### Predicting Innovation Hope

H1, 2, 4, and 7 describe the predictors of hope: innovation attributes (H1), recent malaria illness (H2), collective efficacy (H4), and innovation advocacy (H7). To explore these predictions, innovation hope was regressed onto innovation attributes, recent malaria experience (effect coded as 1 = sick in the past two weeks, −1 = not sick), collective efficacy, and innovation advocacy using OLS. In a second regression, we specified a spatial lag model that used maximum likelihood model estimation and included an explicit spatial lag term for hope with a distance weighting (see Table 2). The diagnostic tests for spatial dependence in the OLS model were not statistically significant: Moran’s *I* = −0.03, *p* = .66, Lagrange multiplier (lag) = .00, *p* = .99, Lagrange multiplier (error) = .30, *p* = .59. The small, insignificant Moran’s *I* for the OLS model indicated virtually no spatial autocorrelation of the residuals. The simple Lagrange multiplier tests showed no evidence of a missing spatially lagged dependent variable (lag test) or correlated errors (error test). In addition, a LL ratio test was not statistically significant, LL *ratio* (1, 118) = 0.54, *p* = .46, which indicated that the spatial lag model did not improve the fit beyond the OLS model. The answer to RQ1, then, was that innovation hope showed no spatial dependence.

**Table 2.** Regression estimates predicting innovation hope (N = 119)

	OLS Estimation			Spatial Lag Model		
	*b*	*SE*	*t*	*b*	*SE*	*z*
Collective efficacy	0.02	0.10	0.24	0.02	0.09	0.20
Innovation attributes	0.62	0.10	6.03*	0.62	0.10	6.26*
Recent malaria illness	−0.14	0.08	−1.82†	−0.13	0.07	−1.78†
Innovation advocacy	0.00	0.04	0.06	0.00	0.04	−0.03
W_Innovation hope	–	–	–	−0.12	0.15	−0.79
Adjusted *R*^2^	.29*			.32*		
Akaike info criterion *(AIC)*	221.64			223.10		
Log likelihood	−105.82			−105.55		

*Notes*. “W” identifies the spatial lag term. Model for the OLS estimate was statistically significant, *F*(5, 114) = 12.95, *p* <.01, adjusted *R*^2^ =.29. The spatial lag model was based on a distance-based spatial weights matrix (using a 60 m threshold).

*p <.05, † *p* <.10.

In both models, collective efficacy and innovation advocacy were not statistically significant predictors of innovation hope. Innovation attributes were statistically significant: better perceptions of the innovation’s attributes predicted stronger innovation hope. Recent malaria illness showed a statistical trend: recent illness predicted less innovation hope. Together, the findings provided support for H1, weak support for H2, and no support for H4 or H7.

#### Collective Efficacy

H3 predicted a positive relationship between perceptions of neighborhood storytelling and collective efficacy. Collective efficacy was regressed onto neighborhood storytelling, using the spatial lag model (LL = −98.97, AIC = 203.93). The *z* value for the spatial autoregressive coefficient for neighborhood storytelling was not statistically significant, *z* = −0.41, *p* = .68, *unstandardized coefficient* = −0.06, *SE* = 0.16, indicating that there was not spatial clustering. The *z* value for neighborhood storytelling was statistically significant, *z* = 5.31, *p* < .001, *unstandardized coefficient* = 0.39, *SE* = 0.07; H3 was supported.

#### Predicting Innovation Advocacy

H5 and H6 together describe three predictors of advocacy: recent malaria (H5), innovation attributes (H6), and collective efficacy (H6). To explore these predictions, innovation advocacy was regressed onto recent malaria experience (effect coded), innovation attributes, and collective efficacy using an OLS model. In a second regression, we specified a spatial lag model that used maximum likelihood model estimation and included an explicit spatial lag term for innovation advocacy on a distance-based spatial weights matrix (using a 60 m threshold, see Table 3).

**Table 3.** Regression estimates predicting innovation advocacy (N = 119)

	OLS Estimation			Spatial Lag Model		
	*b*	*SE*	*t*	*b*	*SE*	*z*
Collective efficacy	0.59	0.20	2.96*	0.57	0.19	2.99*
Innovation attributes	0.68	0.21	3.21*	0.64	0.20	3.19*
Recent malaria illness	0.34	0.16	2.09*	0.32	0.15	2.07*
W_Innovation advocacy				−0.34	0.16	−2.08*
adjusted *R*^2^	.18*			.24*		
Akaike info criterion *(AIC)*	403.02			400.79		
Log likelihood	−197.51			−195.40		

*Notes*. “W” identifies the spatial lag term. Model for the OLS estimate was statistically significant, *F*(4, 115) = 9.54, *p* <.01, adjusted *R*^2^ =.18. The spatial lag model was based on a distance matrix.

*p <.05.

The diagnostic tests for spatial dependence in the OLS model were statistically significant: Global Moran’s *I* = −0.12, *p* < .05, Lagrange multiplier (lag) = 5.32, *p* < .05, Lagrange multiplier (error) = 4.72, *p* < .05. This result indicated spatial autocorrelation of the OLS residuals, introducing bias and inefficiency. The simple Lagrange multiplier tests showed evidence of a missing spatially lagged dependent variable (lag test) and correlated errors (error test). In addition, a LL ratio test was statistically significant, LL *ratio* (1, 118) = 4.23, *p* < .05, which indicated that the spatial lag model did improve the fit beyond the OLS model. The answer to RQ2, then, was that innovation advocacy showed spatial dependence.

The spatial autoregressive coefficient for innovation advocacy was statistically significant and negative, indicating dispersion: advocators were near non-advocate neighbors. The coefficients for innovation attributes, recent malaria illness, and collective efficacy were statistically significant: better perceptions of the innovation’s attributes, greater perceived collective efficacy, and recent malaria illness predicted more innovation advocacy. The findings supported H5 and H6.

## Discussion

This study explored community responses to a communal innovation: an innovation attempting to address a social problem, through wide-spread adoption, that was introduced and installed through a community engagement. Drawing upon CIT (Kim & Ball-Rokeach, 2006b; Kim & Ball‐Rokeach, 2006a) and theories of discrete emotions (e.g., Lazarus, 1991), we explored how appraisals of the innovation’s attributes and collective efficacy fostered hope about the innovation’s ability to decrease malaria and interpersonal advocacy about the innovation. The results showed that innovation hope was predicted by appraisals of the innovation attributes: those who perceived the innovation as having greater relative advantages, compatibility, simplicity, and observability were more hopeful. The results showed that better appraisals of the innovation’s attributes, greater perceived collective efficacy, and recent malaria illness predicted more innovation advocacy. Of note, innovation advocacy was spatially clustered: It presented dispersion, and the local spatial autocorrelation predicted advocacy above and beyond the other predictors. The dispersion for innovation advocacy suggests complementarity or compensation, in which advocators appeared near quiet people (i.e., listeners).

### Theoretical Implications

The findings supported many claims in CIT (Kim & Ball-Rokeach, 2006b; Kim & Ball‐Rokeach, 2006a). According to CIT, people may internalize neighborhood storytelling into their everyday lives, leading to positive perceptions, such as their community’s collective efficacy. Collective efficacy, then, becomes a positive force shaping productive actions that affect collective outcomes through increased dialogue about specific actions. More neighborhood storytelling predicted stronger collective efficacy, and stronger collective efficacy predicted greater personal innovation advocacy. These findings support CIT’s framework (Kim & Ball-Rokeach, 2006b; Kim & Ball‐Rokeach, 2006a), in which the community’s fabric of storytelling supports local storytelling agents through collective efficacy.

CIT (Kim & Ball-Rokeach, 2006b; Kim & Ball‐Rokeach, 2006a) also explicitly considers the social environment fostering local storytelling, which is typically studied as a phenomenon of spatial clustering. CIT presumes that neighbors influence each other, and this social influence shapes outcomes above and beyond individual predictors of action. Innovation advocacy, in particular, was spatially clustered, but the pattern was dispersion. This suggests that local storytelling is not equal across neighbors, but as some people advocate, their neighbors become listeners; this asymmetrical pattern to communication is not unusual (e.g., Bavelas et al., 2000, 2002). Those who had a stronger belief in the innovation, stronger confidence in their community’s collective efficacy, and recent experiences with malaria were the most likely to advocate for the innovation to others. These predictors may provide individuals the motivation and support to speak first, leaving others to listen. These findings speak to recent efforts to theorize about communicative inequities that focus on opportunities to be heard in community problem-solving processes (e.g., Dutta, 2018). In addition to opportunity, there may be a first-mover advantage that may have a silencing effect.

In contrast to advocacy, innovation hope showed no spatial clustering and was not predicted by collective efficacy. Hope was intrapersonal. Innovation hope was strongly predicted by perceptions of the innovation’s attributes: how it fulfills the promise for a better future of less malaria. In a situation in which the problem, malaria, was perceived by all the participants as important, we argued that appraisals of the innovation described in diffusion research (relative advantage, compatibility, complexity, and observability; Rogers, 2003) matched those predicted to evoke hope (Chadwick, 2015; Lazarus, 1991; Roseman, 1991), and should evoke hope. The findings supported this prediction and, overall, suggest that innovations may be a promising context in which to study hope.

In contrast to recent studies of hope appeals (Nabi & Myrick, 2018), in our study, efficacy was unrelated to hope. One possibility is that only self-efficacy, but not collective efficacy, predicts hope. A different possibility is that it is not ability, but imagining a better future that generates hope.

### Limitations and Future Research

The study’s findings are limited by the design and context. The cross-sectional design allowed us to capture the personal and social predictors of hope within a community. It does not provide insights into what may be a dynamic communication process, in which neighbors take turns advocating and listening. Theorizing about CIT would benefit from considering whether dispersion in storytelling is problematic or a normal feature of community life. It may be beneficial for storytelling communities to have a dynamic give and take; or a cluster of stable advocates may be evidence of a few individuals controlling the conversation (Rogers, 2003). In addition, the innovation studied herein had unique features: it was for a wide-spread health condition, it was in its trial phase, and the respondents were active participants in its trial. Indeed, the town hall, for example, provided an opportunity to bring together different types of storytelling agents, not just the micro-level residents of the community, but also the meso-level community leaders and macro-level representatives from the SET innovation team, which may connect the neighborhood storytelling network in particular ways (Matsaganis, Golden, & Scott, 2014). It may be that some opportunities for neighborhood storytelling, such as town-hall meetings, play critical roles in shaping not just the presence of community engagement, such as interpersonal advocacy, but also its spatial pattern. The study should be replicated with other communal innovations at different stages of adoption.

## Conclusion

For a trial technology that promises a better future, there may be a delay between the use of the innovation and its promised outcomes. In addition, for some innovations, like the one studied herein, a better future may rely on community members acting to benefit the community. For slow-moving innovations, hope and problem-solving may encourage communities to give an innovation the time it needs to fulfill its promise. Furthermore, for such projects, it may be particularly important to convey explicitly and clearly how the innovation fulfills a promise for a better future, because such information promotes the appraisals associated with hope and provides critical information for problem-solving communities to make informed decisions.

## References

[cit0001] Adhikari, B., James, N., Newby, G., Seidlein, L., White, N. J., Day, N. P., … Cheah, P. Y. (2016). Community engagement and population coverage in mass anti-malarial administrations: A systematic literature review. *Malaria Journal*, 15(1), 523–544. doi:10.1186/s12936-016-1593-y 27806717 PMC5093999

[cit0002] Anselin, L. (1999). The future of spatial analysis in the social sciences. *Geographic Information Sciences*, 5, 67–76. doi:10.1080/10824009909480516

[cit0003] Anselin, L. (2003). Spatial externalities, spatial multipliers, and spatial econometrics. *International Regional Science Review*, 26(2), 153–166. doi:10.1177/0160017602250972

[cit0004] Anselin, L., Syabri, I., & Kho, Y. (2006). GeoDa: An introduction to spatial data analysis. *Geographical Analysis*, 38(1), 5–22. doi:10.1111/j.0016-7363.2005.00671.x

[cit0005] Averill, J. R., Catlin, G., & Chon, K. K. (1990). *Rules of hope*. New York, NY: Springer-Verlag.

[cit0006] Bandura, A. (2000). Exercise of human agency through collective efficacy. *Current Directions in Psychological Science*, 9(3), 75–78. doi:10.1111/1467-8721.00064

[cit0007] Bavelas, J. B., Coates, L., & Johnson, T. (2000). Listeners as co-narrators. *Journal of Personality and Social Psychology*, 79(6), 941–952. doi:10.1037/0022-3514.79.6.941 11138763

[cit0008] Bavelas, J. B., Coates, L., & Johnson, T. (2002). Listener responses as a collaborative process: The role of gaze. *Journal of Communication*, 52(3), 566–580. doi:10.1111/j.1460-2466.2002.tb02562.x

[cit0009] Boster, F. J., Carpenter, C. J., Andrews, K. R., & Mongeau, P. A. (2012). Employing interpersonal influence to promote behavioral change. *Health Communication*, 27(4), 399–407. doi:10.1080/10410236.2011.595771 21957941

[cit0010] Bruininks, P., & Malle, B. F. (2005). Distinguishing hope from optimism and related affective states. *Motivation and Emotion*, 29(4), 324–352. doi:10.1007/s11031-006-9010-4

[cit0011] Chadwick, A. E. (2015). Toward a theory of persuasive hope: Effects of cognitive appraisals, hope appeals, and hope in the context of climate change. *Health Communication*, 30(6), 598–611. doi:10.1080/10410236.2014.916777 25297455

[cit0012] Dearing, J. W. (2009). Applying diffusion of innovation theory to intervention development. *Research on Social Work Practice*, 19(5), 503–518. doi:10.1097/01.PHH.0000311886.98627.b7 20976022 PMC2957672

[cit0013] Dutta, M. J. (2018). Culture-centered approach in addressing health disparities: Communication infrastructures for subaltern voices. *Communication Methods and Measures*, 12(4), 239–259. doi:10.1080/19312458.2018.1453057

[cit0014] Fischer, M., & Getis, A. (2010). *Handbook of applied spatial analysis*. Berlin, Germany: Springer.

[cit0015] Gonzalez, R., & Griffin, D. (2000). On the statistics of interdependence: Treating dyadic data with respect. In W. Ickes & S. Duck (Eds.), *The social psychology of personal relationships* (pp. 181–213). New York, NY: Wiley.

[cit0016] Hatfield, E., Cacioppo, J. T., & Rapson, R. L. (1993). Emotional contagion. *Current Directions in Psychological Science*, 2(3), 96–100. doi:10.1111/1467-8721.ep10770953

[cit0017] Hawley, W. A., Phillips-Howard, P. A., Ter Kuile, F. O., Terlouw, D. J., Vulule, J. M., Ombok, M., … Hightower, A. W. (2003). Community-wide effects of permethrin-treated bed nets on child mortality and malaria morbidity in western Kenya. *The American Journal of Tropical Medicine and Hygiene*, 68(4_suppl), 121–127. doi:10.4269/ajtmh.2003.68.121 12749495

[cit0018] Kenny, D. A., Mannetti, L., Pierro, A., Livi, S., & Kashy, D. A. (2002). The statistical analysis of data from small groups. *Journal of Personality and Social Psychology*, 83(1), 126–137. doi:10.1037/0022-3514.83.1.126 12088122

[cit0019] Killeen, G. F., & Moore, S. J. (2012). Target product profiles for protecting against outdoor malaria transmission. *Malaria Journal*, 11(1), 17–28. doi:10.1186/1475-2875-11-17 22236388 PMC3298720

[cit0020] Kim, Y. C., & Ball-Rokeach, S. J. (2006b). Community storytelling network, neighborhood context, and civic engagement: A multilevel approach. *Human Communication Research*, 32(4), 411–439. doi:10.1111/j.1468-2958.2006.00282.x

[cit0021] Kim, Y. C., & Ball‐Rokeach, S. J. (2006a). Civic engagement from a communication infrastructure perspective. *Communication Theory*, 16(2), 173–197. doi:10.1111/j.1468-2885.2006.00267.x

[cit0022] Kim, Y. C., Moran, M. B., Wilkin, H. A., & Ball-Rokeach, S. J. (2011). Integrated connection to neighborhood storytelling network, education, and chronic disease knowledge among African Americans and Latinos in Los Angeles. *Journal of Health Communication*, 16(4), 393–415. doi:10.1080/10810730.2010.546483 21302173

[cit0023] Kim, Y.-C., & Kang, J. (2010). Communication, neighborhood belonging and household hurricane preparedness. *Disasters*, 34(2), 470–488. doi:10.1111/j.1467-7717.2009.01138.x 19878261

[cit0024] Knols, B. G., Farenhorst, M., Andriessen, R., Snetselaar, J., Suer, R. A., Osinga, A. J., … Kessy, S. T. (2016). Eave tubes for malaria control in Africa: An introduction. *Malaria Journal*, 15(1), 404–411. doi:10.1186/s12936-016-1452-x 27515306 PMC4982263

[cit0025] Lazarus, R. S. (1991). *Emotion and adaptation*. New York, NY: Oxford University Press.

[cit0026] Lazarus, R. S. (1999). Hope: An emotion and a vital coping resource against despair. *Social Research*, 653-678.

[cit0027] Matsaganis, M. D. (2008). Rediscovering the communication engine of neighborhood effects: How the interaction of residents and community institutions impacts health literacy and how it can be leveraged to improve health care access (Doctoral dissertation). ProQuest Dissertations & Theses database. (UMI No. 3344419).

[cit0028] Matsaganis, M. D., Golden, A. G., & Scott, M. (2014). Communication infrastructure theory and reproductive health disparities: Enhancing storytelling network integration by developing interstitial actors. *International Journal of Communication*, 8, 1495–1515.

[cit0029] Matsaganis, M. D., & Wilkin, H. A. (2015). Communicative social capital and collective efficacy determinants of access to health-enhancing resources in residential communities. *Journal of Health Communication*, 20(4), 377–386. doi:10.1080/10810730.2014.927037 25529115

[cit0030] Nabi, R. L. (2010). The case for emphasizing discrete emotions in communication research. *Communication Monographs*, 77(2), 153–159. doi:10.1080/03637751003790444

[cit0031] Nabi, R. L., & Myrick, J. G. (2018). Uplifting fear appeals: Considering the role of hope in fear-based persuasive messages. *Health Communication*, 1–12. doi:10.1080/10410236.2017.1422847 29313717

[cit0032] Parkinson, B. (2011). Interpersonal emotion transfer: Contagion and social appraisal. *Social and Personality Psychology Compass*, 5(7), 428–439. doi:10.1111/j.1751-9004.2011.00365.x

[cit0033] Parkinson, B., & Simons, G. (2009). Affecting others: Social appraisal and emotion contagion in everyday decision making. *Personality & Social Psychology Bulletin*, 35(8), 1071–1084. doi:10.1177/0146167209336611 19474455

[cit0034] Rogers, E. M. (2003). *Diffusion of innovations* (5th ed. ed.). New York, NY: Free Press.

[cit0035] Roseman, I. J. (1991). Appraisal determinants of discrete emotions. *Cognition & Emotion*, 5(3), 161–200. doi:10.1080/02699939108411034

[cit0036] Roseman, I. J. (2001). A model of appraisal in the emotion system: Integrating theory, research, and applications. In K. R. Scherer, A. Schorr, & T. Johnstone (Eds.), *Appraisal processes in emotion: Theory, methods, research* (pp. 68–91). New York, NY: Oxford University Press.

[cit0037] Sahan, K., Pell, C., Smithuis, F., Phyo, A. K., Maung, S. M., Indrasuta, C., … Cheah, P. Y. (2017). Community engagement and the social context of targeted malaria treatment: A qualitative study in Kayin (Karen) State, Myanmar. *Malaria Journal*, 16(1), 75–85. doi:10.1186/s12936-017-1718-y 28196536 PMC5310060

[cit0038] Sampson, R. J., Raudenbush, S. W., & Earls, F. (1997). Neighborhoods and violent crime: A multilevel study of collective efficacy. *Science*, 277(5328), 918–924. doi:10.1126/science.277.5328.918 9252316

[cit0039] Smith, P. G., Morrow, R. H., & Ross, D. A. (2015). *Field trials of health interventions: A tool box*. Oxford, UK: Oxford University Press. 26225404

[cit0040] Snyder, C. R. (2000). *Handbook of Hope: Theory, measures, and applications*. San Diego, CA: Academic.

[cit0041] Sternberg, E. D., Cook, J., Alou, L. P. A., Aoura, C. J., Assi, S. B., Doudou, D. T., … Worrall, E. (2018). Evaluating the impact of screening plus eave tubes on malaria transmission compared to current best practice in central Côte d’Ivoire: A two armed cluster randomized controlled trial. *BMC Public Health*, 18(1), 894–912. doi:10.1186/s12889-018-5746-5 30021543 PMC6052618

[cit0042] Stotland, E. (1969). *The psychology of hope*. San Francisco, CA: Jossey-Bass.

[cit0043] Sullins, E. S. (1991). Emotional contagion revisited: Effects of social comparison and expressive style on mood convergence. *Personality & Social Psychology Bulletin*, 17(2), 166–174. doi:10.1177/014616729101700208

[cit0044] Waite, J. L., Lynch, P. A., & Thomas, M. B. (2016). Eave tubes for malaria control in Africa: A modelling assessment of potential impact on transmission. *Malaria Journal*, 15(1), 449–459. doi:10.1186/s12936-016-1505-1 27590602 PMC5009529

[cit0045] Walter, N., Robbins, C., Murphy, S. T., & Ball-Rokeach, S. J. (2017). The weight of networks: The role of social ties and ethnic media in mitigating obesity and hypertension among Latinas. *Ethnicity & Health*, 1–14. doi:10.1080/13557858.2017.1373071 PMC6089665 28862887

[cit0046] Webb, D. (2007). Modes of hoping. *History of the Human Sciences*, 20(3), 65–83. doi:10.1177/0952695107079335

[cit0047] Whittaker, M., & Smith, C. (2015). Reimagining malaria: Five reasons to strengthen community engagement in the lead up to malaria elimination. *Malaria Journal*, 14(1), 410–416. doi:10.1186/s12936-015-0931-9 26474852 PMC4608300

[cit0048] Wilkin, H. A. (2013). Exploring the potential of communication infrastructure theory for informing efforts to reduce health disparities. *Journal of Communication*, 63(1), 181–200. doi:10.1111/jcom.12006

[cit0049] Wilkin, H. A., Katz, V. S., Ball-Rokeach, S. J., & Hether, H. J. (2015). Communication resources for obesity prevention among African American and Latino residents in an urban neighborhood. *Journal of Health Communication*, 20(6), 710–719. doi:10.1080/10810730.2015.1018559 25928242

[cit0050] Wilkin, H. A., Stringer, K. A., O’Quin, K., Montgomery, S. A., & Hunt, K. (2011). Using communication infrastructure theory to formulate a strategy to locate “hard-to-reach” research participants. *Journal of Applied Communication Research*, 39(2), 201–213. doi:10.1080/00909882.2011.556140

[cit0051] Wlodarczyk, A., Basabe, N., Páez, D., & Zumeta, L. (2017). Hope and anger as mediators between collective action frames and participation in collective mobilization: The case of 15-M. *Journal of Social and Political Psychology*, 5(1), 200–223. doi:10.5964/jspp.v5i1.471

[cit0052] World Health Organization. (2017). *World malaria report 2017*. Geneva, CH: WHO.

